# Northern forest winters have lost cold, snowy conditions that are important for ecosystems and human communities

**DOI:** 10.1002/eap.1974

**Published:** 2019-08-07

**Authors:** Alexandra R. Contosta, Nora J. Casson, Sarah Garlick, Sarah J. Nelson, Matthew P. Ayres, Elizabeth A. Burakowski, John Campbell, Irena Creed, Catherine Eimers, Celia Evans, Ivan Fernandez, Colin Fuss, Thomas Huntington, Kaizad Patel, Rebecca Sanders‐DeMott, Kyongho Son, Pamela Templer, Casey Thornbrugh

**Affiliations:** ^1^ Earth Systems Research Center Institute for the Study of Earth, Oceans, and Space University of New Hampshire 8 College Road Durham New Hampshire 03824 USA; ^2^ Department of Geography University of Winnipeg 515 Portage Avenue Winnipeg Manitoba R3B 2E9 Canada; ^3^ Hubbard Brook Research Foundation 30 Pleasant Street Woodstock Vermont 05091 USA; ^4^ School of Forest Resources University of Maine 5755 Nutting Hall Orono Maine 04469 USA; ^5^ Department of Biological Sciences Dartmouth College 78 College Street Hanover New Hampshire 03755 USA; ^6^ USDA Forest Service, Northern Research Station 271 Mast Road Durham New Hampshire 03824 USA; ^7^ School of Environment and Sustainability University of Saskatchewan 117 Science Place Saskatoon Saskatchewan S7N 5C8 Canada; ^8^ School of the Environment Trent University 1600 West Bank Drive Peterborough Ontario K9L 0G2 Canada; ^9^ Department of Natural Science Paul Smith's College Freer Science Building, 7833 New York 30 Paul Smiths New York 12970 USA; ^10^ Climate Change Institute and School of Forest Resources University of Maine Deering Hall Orono Maine 04469 USA; ^11^ Cary Institute of Ecosystem Studies 2801 Sharon Turnpike Millbrook New York 12545 USA; ^12^ New England Water Science Center United States Geological Survey 196 Whitten Road Augusta Maine 04330 USA; ^13^ Pacific Northwest National Laboratory Biological Sciences Division P.O. Box 999 Richland Washington 99352 USA; ^14^ Research Foundation of the City University of New York 230 West 41st Street New York New York 10036 USA; ^15^ Department of Biology Boston University 5 Cummington Mall Boston Massachusetts 02215 USA; ^16^ United South and Eastern Tribes, Inc. 711 Stewarts Ferry Pike # 100 Nashville Tennessee 37214 USA; ^17^ DOI Northeast & Southeast Climate Adaptation Science Centers Morrill Science Center University of Massachusetts, Amherst 611 North Pleasant Street Amherst Massachusetts 01003 USA

**Keywords:** climate change, indicator, northern forest, snow, temperature, winter

## Abstract

Winter is an understudied but key period for the socioecological systems of northeastern North American forests. A growing awareness of the importance of the winter season to forest ecosystems and surrounding communities has inspired several decades of research, both across the northern forest and at other mid‐ and high‐latitude ecosystems around the globe. Despite these efforts, we lack a synthetic understanding of how winter climate change may impact hydrological and biogeochemical processes and the social and economic activities they support. Here, we take advantage of 100 years of meteorological observations across the northern forest region of the northeastern United States and eastern Canada to develop a suite of indicators that enable a cross‐cutting understanding of (1) how winter temperatures and snow cover have been changing and (2) how these shifts may impact both ecosystems and surrounding human communities. We show that cold and snow covered conditions have generally decreased over the past 100 years. These trends suggest positive outcomes for tree health as related to reduced fine root mortality and nutrient loss associated with winter frost but negative outcomes as related to the northward advancement and proliferation of forest insect pests. In addition to effects on vegetation, reductions in cold temperatures and snow cover are likely to have negative impacts on the ecology of the northern forest through impacts on water, soils, and wildlife. The overall loss of coldness and snow cover may also have negative consequences for logging and forest products, vector‐borne diseases, and human health, recreation, and tourism, and cultural practices, which together represent important social and economic dimensions for the northern forest region. These findings advance our understanding of how our changing winters may transform the socioecological system of a region that has been defined by the contrasting rhythm of the seasons. Our research also identifies a trajectory of change that informs our expectations for the future as the climate continues to warm.

## Introduction

Winter is an understudied but key period for the socioecological system of northeastern North American forests (Campbell et al. 2005). Low air temperatures promote forest health by killing insect pests that might otherwise proliferate during the growing season (Skinner et al. 2003, Dukes et al. 2009, Weed et al. 2013). At the same time, a deep snowpack insulates soil (Hardy et al. 2001, Decker et al. 2003, Tatariw et al. 2017), thereby providing subnivean refugia to burrowing animals (Penczykowski et al. 2017). An insulating snowpack also prevents the freezing of roots (Cleavitt et al. 2008, Comerford et al. 2013) and microbes (Haei et al. 2012), both of which can alter soil nutrient cycling and hydrological processes during winter and the ensuing growing season (Groffman et al. 2001, Brooks et al. 2011, Campbell et al. 2014, Creed et al. 2015).

Sub‐zero air temperatures and deep snow cover are as important to the region's economy as they are to its ecosystem functions. Timber harvesting on wet sites and bottomlands often occurs in winter when soils are either snow covered or frozen, which minimizes soil disturbance (Wolf et al. 2008, Rittenhouse and Rissman 2015). Maple sugaring depends on sufficiently cold winters that permit below‐freezing nights followed by above‐freezing days; if temperatures are not cold enough, the quantity of sap is reduced (Skinner et al. 2010, Houle et al. 2015). The ski industry requires a deep and persistent snowpack to attract skiers (Hamilton et al. 2007, Dawson and Scott 2013), and mountain communities rely on these snow sport visitors to generate tourism revenue (Hagenstad et al. 2018). Winter is also culturally important to the region, playing a prominent role in its history (Brooks 2017, Wickman 2017) and in influencing the current ways in which people interact with the outdoors through activities such as skiing, snowshoeing, ice skating, snowmobiling, and ice fishing (Scott et al. 2008, Dawson et al. 2013). Cold, snowy winters help support iconic wildlife that are also important to tourism, fishing, and hunting, including fishing and hunting for subsistence and other cultural uses by Indigenous peoples (Norton‐Smith et al. 2016, Penczykowski et al. 2017). In addition, sufficiently cold winter air temperatures and deep snowpack can limit the spread of vector‐borne diseases such as Lyme disease and West Nile virus, and thus are important to human health (Ogden et al. 2014, Beard et al. 2016).

A growing awareness of the importance of the winter season to ecosystems and surrounding communities has inspired several decades of research across the northeastern United States, eastern Canada, and other mid‐ and high‐latitude ecosystems around the globe (Bowman and Seastedt 2001, Barnett et al. 2005, Campbell et al. 2005, Mote et al. 2005, Scott et al. 2008, Brooks et al. 2011, Pradhanang et al. 2013, Mankin et al. 2015, Rittenhouse and Rissman 2015, Lesk et al. 2017, Penczykowski et al. 2017, Hagenstad et al. 2018). This research focus has become increasingly important as winters are changing. Although annual average air temperatures are increasing across northeastern North America (Vincent et al. 2015, Vose et al. 2017), winter air temperatures have been warming at faster rates (Hayhoe et al. 2007, Donat et al. 2013, Vincent et al. 2015), with trends expected to persist throughout the century (Hayhoe et al. 2007, Lemmen et al. 2008). This loss of coldness has impacted regional snow cover through a greater proportion of precipitation falling as rain or sleet instead of snow (Huntington et al. 2004, Feng and Hu 2007) and has resulted in earlier snowmelt (Hodgkins et al. 2003, Hodgkins and Dudley 2006, Matonse et al. 2011, Zion et al. 2011, Pradhanang et al. 2013). The snowpack in northeastern North America has already decreased both in depth and duration (Hodgkins and Dudley 2006, Burakowski et al. 2008, Campbell et al. 2010), and this pattern is expected to continue (Hodgkins and Dudley 2006, Frumhoff et al. 2008, Campbell et al. 2010).

There is an emerging consensus that winter is an important period for the functioning of both forested ecosystems and human communities, and that winter temperature, precipitation, and snow cover are changing with climate change. However, we lack a synthetic understanding of how these changes in winter climate may impact cold‐season ecological processes and the social and economic activities the ecosystem supports. Previous work examining the effects of winter climate change on ecological processes has typically occurred at single sites or over short time spans that range from 1 to 3 years (Groffman et al. 2001, 2011, Contosta et al. 2011, Casson et al. 2012, Templer et al. 2012, Kurian et al. 2013, Bergeron and Pekins 2014, Campbell et al. 2014, Fuss et al. 2016, Patel et al. 2018, Sorensen et al. 2018) or in laboratory or mesocosm simulations (Zhu et al. 2002, Reinmann et al. 2012), and are difficult to scale over larger areas and longer periods (Blume‐Werry et al. 2016). Prior studies that have included a broader scope in space and time have typically examined minimum temperature and snow cover duration as indicators of change in winter climate (Dyer and Mote 2006, Donat et al. 2013). These studies have not explicitly tested how changes in winter climate might affect both social and ecological systems. In this study, we take advantage of 100 years of meteorological observations across the northeastern United States and eastern Canada to develop a suite of indicators that enable a cross‐cutting understanding of (1) how winter temperatures and snow cover have changed across the northern forest region of northeastern North America and (2) how these shifts may impact both ecosystems and surrounding human communities. We couple our analysis of winter climate change indicators with past research examining ecological, social, or economic attributes of the region to portray how winter climate change impacts the socioecological system of the northern forest.

## Methods

### Study area and data sources

**Figure 1:**
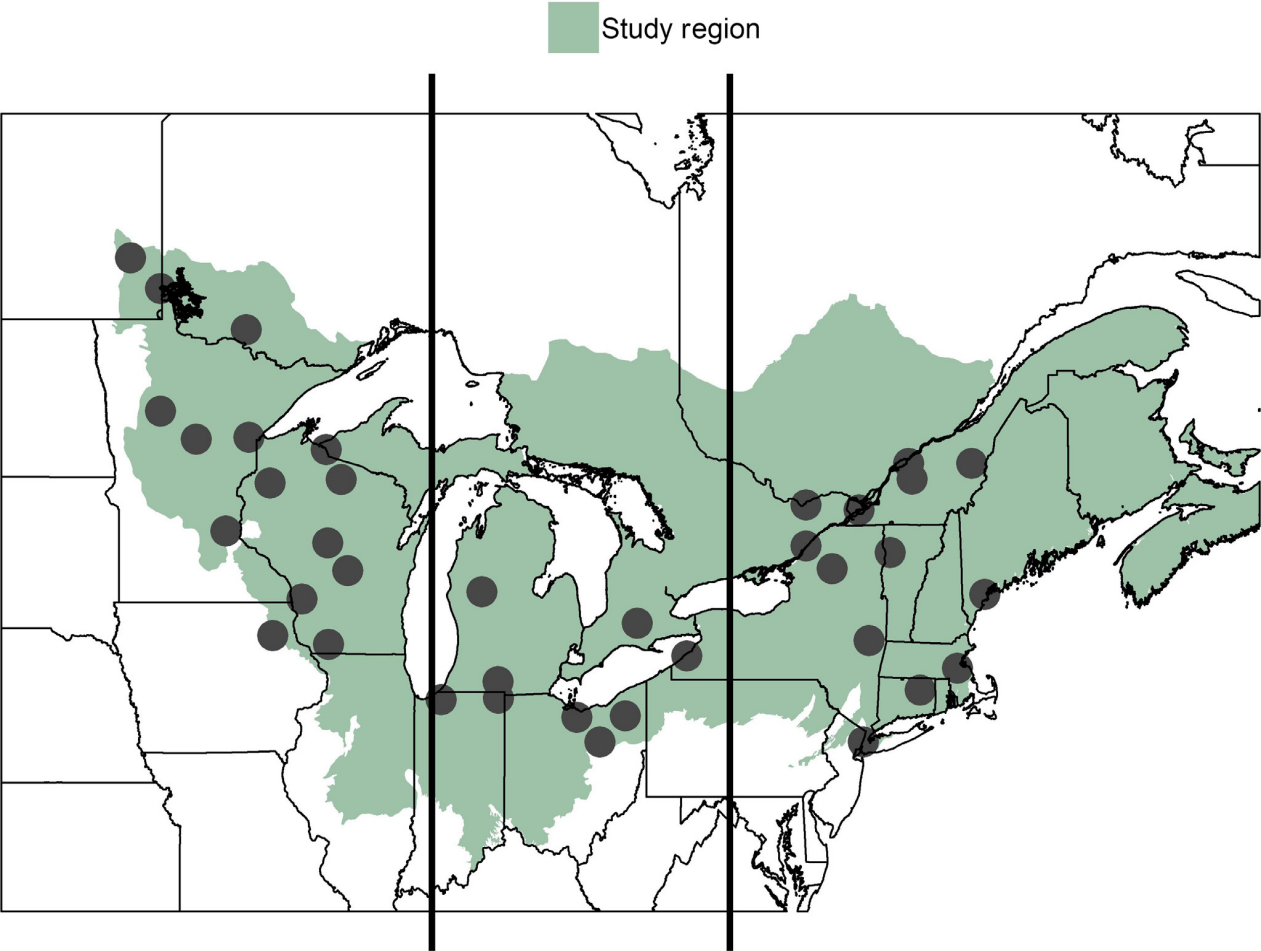
Map of study area. Points indicate locations of weather stations included in the analysis located within as defined by Level II ecoregion classifications. Vertical lines show three subregions delineated for the purpose of examining broad spatial differences among trends.

This study focuses on the northern forest region of the northeastern North America, which was identified using the hierarchical ecoregion classification system of Omernik and Griffith (2014). Level II (subcontinental) classification systems were used to delineate the study area that consisted of northern and eastern forest types (Fig. 1). Although this area encompasses agricultural, urban, and other types of land cover, forests are the dominant land cover type across much of the study domain, comprising ~50–80% of land cover in most of the states and provinces where stations were located (Wulder et al. 2004, Nowak and Greenfield 2012). Within the study area, weather stations were selected from the National Climate Data Archive of Environment Canada (NCDAEC; Mekis and Vincent 2011, Vincent et al. 2012) and the United States Historical Climatology Network (USHCN; Easterling et al. 1999, Williams et al. 2006), a subset of the National Oceanic and Atmospheric Administration's Cooperative Observer Program Network selected for spatial coverage, record length and completeness, and historical stability. Both the NCDAEC and the USHCN provide high‐quality data sets tested and adjusted for homogeneities and biases that can arise from issues such as station moves, differences in measurement practices among stations, changes in measurement protocols over time, urban warming, and other non‐climatic influences. Only weather stations with at least 100 years of precipitation and/or temperature data were included in the analysis to facilitate trend detection (Barnett et al. 1999). To maximize the number of sites we were able to include with this 100‐year record, we neither targeted sites in more forested areas nor removed sites closer to urban centers. For the U.S. weather stations, we obtained daily minimum and maximum temperatures (°C), total precipitation (mm liquid), total snowfall (mm solid), and snow depth (mm solid) data from the National Climatic Data Center (NCDC) Climate Data portal (*available online*).^1^ For Canadian stations, daily minimum and maximum temperatures (°C), total precipitation (mm liquid), and total snowfall (mm snow water equivalent or SWE) were downloaded from the NCDAEC (*available online*) and snow depth (mm solid) was obtained from the Adjusted and Homogenized Canadian Climate Data (Vincent et al. 2012).^2^ Although data sources from both the United States and Canada underwent extensive quality control prior to public release, we implemented additional pre‐processing procedures to (1) screen stations for record completeness both within a given winter and across the measurement period; and (2) gap‐fill snow data (see Supporting Information for more details on data pre‐processing). We defined winter as occurring between 1 November and 31 May to capture both the period of biological dormancy between autumn senescence and spring leaf‐out, as well as early and late freezing and snow events across the northern forest study area (Kunkel et al. 2009). We recognize that this period likely extended into the growing season in stations located in more southerly sites and may have omitted early and late season frosts at more northern stations. Nevertheless, this definition of the “dormant season” allowed us to characterize how winters are changing in ways that are not typically captured in studies that focus only on the meteorological period of winter (December, January, and February in the northern hemisphere; Trenberth 1983). Overall, 37 stations were retained for development and testing of winter climate change indicators (Appendix S1; Table S1).

We did not remove stations that failed to pass snow depth criteria when implementing record completeness screening given the incompleteness of snow data within the study area. Time series for snow depth records typically began ~1950 for both the Canadian and U.S. data sources, resulting in highly disparate record lengths among variables within a given site. Even measurements made after ~1950 contained many gaps since “missing” snow depth data can arise from observers failing to record snow depth as zero if snow is not on the ground (Kunkel et al. 2009). Thus, we opted to use modeled SWE as opposed to measured snow depth values across the entire study period using a degree‐day snowmelt model (Kokkonen et al. 2006, Buttle 2009, Crossman et al. 2016) implemented in R (R Core Team 2017) using the snow.sim function within the hydromad package (Andrews et al. 2011). Additional details on the parameterization and validation of the model are provided in the Supporting Information.

### Indicators of winter climate change and its impacts

We assessed long‐term changes in winter conditions using a suite of indicators relevant for understanding both climatic changes and their impacts on ecosystems and human communities (Table 1). Some of these indicators were previously defined by the joint World Meteorological Organization Commission on Climatology (CCl) and the Climate Variability and Prediction (CLIVAR) Expert Team on Climate Change Detection and Indices (ETCCDI) to standardize definitions and analyses of climate change (Karl et al. 1999, Brown et al. 2010, Donat et al. 2013). These include Ice Days (daily maximum temperature or *T*_max_ < 0°C), Frost Days (daily minimum temperature or *T*_min_ < 0°C), and Thaw Days (*T*_max_ > 0°C) that represented general indicators of winter climate, where the terms Ice, Frost, and Thaw Days were used to be consistent with existing World Meteorological Organization terminology.

**Table 1:** List of winter climate change indicators, their definition, their relevance to forest ecosystems and/or surrounding communities, and references for prior studies used in developing them

Indicator name	Indicator definition	Social‐ecological relevance	References
Thaw day	*T*_max_ > 0°C	general	Karl et al. (1999); Brown et al. (2010), Donat et al. (2013)
Ice day	*T*_max_ < 0°C	general	Karl et al. (1999), Brown et al. (2010), Donat et al. (2013)
Frost day	*T*_min_ < 0°C	general	Karl et al. (1999), Brown et al. (2010), Donat et al. (2013)
Extreme cold day/pine beetle kill day	*T*_min_ < −18°C	human health, forest health	DeGaetano (1996), Ungerer et al. (1999), Lombardero et al. (2000), Trân et al. (2007)
Hemlock Woolly Adelgid Kill Day	*T*_min_ < −30°C	forest health	Skinner et al. (2003), Tobin et al. (2017)
Snowmaking day/mosquito kill day	*T*_min_ < −5°C (before December 25; before February 28)	recreation and tourism, human health	Scott et al. (2006), Wilson et al. (2018)/Platonov et al. (2008), Rochlin et al. (2013), Ogden et al. (2014)
Snow covered day	snow depth > 0 mm	general	Hayhoe et al. (2007), Burakowski et al. (2008)
Bare ground day	snow depth = 0 mm	general	Hayhoe et al. (2007), Burakowski et al. (2008)
Rain‐on‐snow day	liquid precipitation > 0 mm and snow depth > 0 mm	ecosystem function	Casson et al. (2010, 2012), Crossman et al. (2016)
Bare ground Ice day/frozen ground day	*T*_max_ < 0°C and snow depth = 0 mm	ecosystem function, logging	Groffman et al. (2001), Campbell et al. (2005), Cleavitt et al. (2008), Campbell et al. (2014), Tatariw et al. (2017), Sanders‐DeMott et al. (2018a), Patel et al. (2018)
Bare ground thaw day/mud day	*T*_max_ > 0°C and snow depth = 0 mm	ecosystem function, logging, recreation, and tourism	Stone (2002), Scott et al. (2008), Rittenhouse and Rissman (2015)

*Note*

*T*_min_, minimum temperature; *T*_max_, maximum temperature.

We supplemented these existing indicators with a new set of indicators that could explicitly consider how changing winter temperature, precipitation, snowfall, and snow depth might impact forested ecosystems and surrounding communities. This new set of indicators originated from the scientific literature, scientific experts, and discussions with key stakeholders with whom we have long‐standing partnerships (see Appendix S1 for a fuller description of indicator development based on stakeholder engagement). Indicators were grouped into categories related to coldness, snowpack, or both. For example, temperature‐based indicators relevant for winter recreation and tourism included Snowmaking Days, which represent conditions suitable for making artificial snow. While conditions appropriate for snowmaking change with technological improvements (Appendix S1; Table S2), we elected to use a temperature threshold of *T*_min_ < −5°C for the sake of simplicity. (Scott et al. 2003). For this *T*_min_ < −5°C threshold, we evaluated changes in snowmaking opportunities for two time periods that represent historical visitation patterns of economic importance to ski areas: prior to 25 December and prior to 28 February (Scott et al. 2006, Wilson et al. 2018).

In some cases, we developed multiple winter climate change indicators from a single threshold. For instance, snowmaking temperature thresholds of *T*_min_ < −5°C are also lethal for some invasive, disease‐carrying insects such as the Asian tiger mosquito (*Aedes albopictus* Skuse; Platonov et al. 2008, Rochlin et al. 2013, Ogden et al. 2014). A Snowmaking Day, where *T*_min_ < −5°C, could double as an indicator for both potential winter recreational activities and as a Mosquito Kill Day, which is relevant for understanding how changing winters might impact human health. Likewise, Extreme Cold Days, in which *T*_min_ < −18°C (0°F), can also negatively impact human health through frostbite or hypothermia. At the same time, these Extreme Cold Days can positively affect forest health by preventing the northward advancement of forest insect pests such as the southern pine beetle (*Dendroctonus frontalis* Zimmermann) whose supercooling point (lower lethal temperatures) can range from −14 to −20°C (Ungerer et al. 1999, Lombardero et al. 2000, Trân et al. 2007).

Indicators that featured both temperature and snowpack thresholds included Bare Ground Ice Days and Bare Ground Thaw Days. A Bare Ground Ice Day illustrates a situation where the combination of an absent snowpack plus cold air temperatures might result in soil freezing, microbial and fine root mortality, and nutrient leaching (Groffman et al. 2001, Campbell et al. 2005, 2014, Cleavitt et al. 2008, Tatariw et al. 2017, Patel et al. 2018, Sanders‐DeMott et al. 2018a). By contrast, a Bare Ground Thaw Day exemplifies a scenario in which the absence of snow plus warm air temperatures might result in conditions that reduce access to winter forest harvest sites (Rittenhouse and Rissman 2015), expose hiking trails to erosion risk, and limit opportunities for skiing, snowmobiling, and other winter sports (Scott et al. 2008).

### Statistical analysis

We calculated the frequency with which each indicator occurred for each site by year combination, counting across the entire period from 1 November to 31 May annually to encapsulate the whole dormant season between senescence and leaf‐out while also capturing early‐ and late‐season snowfall and soil frost events. Within sites, we evaluated change over time for each climate variable using the Mann‐Kendall test for trends in time series data (Mann 1945) and determined the rate of change for each variable using nonparametric Sen slope analysis (Sen 1968). To ensure that our data did not exhibit serial correlation, we used the acf function in R to examine the potential autocorrelation of winter climate change indicators for each site and each indicator included in the study. We set the maximum lag between pairs of observations included in the autocorrelation analysis to 30 years, or the length of a climate normal. None of the resulting correlation coefficients from the acf test crossed the upper or lower thresholds for statistical significance (in this case the 95% confidence interval), and thus, we concluded that our data met the independence assumption of the Mann‐Kendall test. Because we calculated the frequency with which each indicator occurred over an annual basis, we assumed that our data did not exhibit seasonal behavior that that would require seasonal Mann‐Kendall and Sen slope analyses.

We examined the regional significance of trends over time using regional Mann‐Kendall and Sen slope analyses. These regional tests are like seasonal Mann‐Kendall and Sen slope analyses (Hirsch et al. 1982, Hirsch and Slack 1984) but use location instead of season as the blocking identifier (Helsel and Frans 2006, Winslow et al. 2015). For each indicator, we used the rkt function (Marchetto et al. 2013) to determine the statistical significance of trends across the entire region. This statistical significance was determined from the two‐sided *P* value that the rkt test provides, while the regional trend was evaluated using the rkt Sen slope output. We set site as the blocking factor and assumed no correlation between blocks.

In addition to examining trends in winter climate change indicators across the entire study area, we divided the northern forest region into three geographic subregions, west, central, and east, to examine broad spatial differences among trends (Fig. 1). For the purpose of this study, sites in the western subregion were west of 87° W, sites in the central subregion were between 78° W and 87° W, and sites in the eastern subregion were east of 78° W. We conducted the same regional Mann‐Kendall and Sen slope analyses on each subregion using the same approach as for the entire northern forest. To gain additional insight into the spatial coherence of trends, we supplemented our regional and subregional statistical analyses with calculations of median, minimum, and maximum Sen slopes where trends were significant based on a Mann‐Kendall test (α = 0.05) within each of these geographic subregions (Table 2). We also determined median, minimum, and maximum numbers of days that each indicator occurred over the whole 100‐year record by subregion (Table 3).

**Table 2:** Summary of statistics demonstrating change over time in winter climate change indicators

Indicator name	West (*n* = 15)					Central (*n* = 9)					East (*n* = 13)				
	No. pos	No. neg	Med slope	Reg slope	Range of slopes	No. pos	No. neg	Med slope	Reg slope	Range of slopes	No. pos	No. neg	Med slope	Reg slope	Range of slopes
Thaw day	5	0	+1.2	0.5	+1.0, +2.1	1	5	−1.2	−0.5	−1.6, +1.7	5	1	+1.8	0.8	−1.3, +2.2
Ice day	2	4	−1.3	−0.4	−2.3, +1.5	6	1	+1.3	0.6	−1.6, +2.0	0	6	−1.4	−1.1	−2.0, −1.0
Frost day	0	10	−1.1	−0.8	−1.7, −0.8	1	3	−1.5	−0.5	−3.8, +1.2	0	13	−1.8	−2.1	−3.5, −1.2
Extreme cold/pine beetle kill day	1	6	−1.4	−0.7	−2.2, +1.2	2	0	+0.6	ns	+0.5, +0.7	0	9	−1.5	−0.5	−1.8, −0.2
Hemlock Woolly Adelgid Kill Day	0	7	−0.5	0.0	−1.1, −0.1	0	0	0.0	ns	0.0, 0.0	0	5	0.0	0.0	−0.3, 0.0
Snowmaking/mosquito kill day (before 28 February)	0	6	−0.9	−0.5	−1.6, −0.8	1	1	+0.1	ns	−1.3, +1.4	0	12	−1.5	−1.6	−2.7, −0.8
Snowmaking/mosquito kill day (before 25 December)	0	9	−1.5	−0.9	−2.2, −0.9	2	0	+1.4	ns	+1.1, +1.8	0	11	−1.6	−1.6	−3.1, −1.0
Snow covered day	0	4	−1.7	−0.8	−2.0, −1.3	2	2	0.0	ns	−2.1, +1.9	0	10	−2.1	−1.9	−4.5, −1.4
Bare ground day	4	0	+1.8	0.7	+1.3, +2.6	1	1	−0.5	−0.5	−2.8, +1.8	7	0	+2.1	1.5	+1.5, +2.8
Rain‐on‐snow day	2	1	+0.1	0.0	0.0, +0.2	2	0	0.0	0.0	0.0, 0.0	0	3	−0.5	0.0	−0.7, +0.2
Bare ground ice/frozen ground day	0	1	0.0	ns	0.0, 0.0	0	2	−0.4	−1.1	−0.5, −0.3	0	0	0.0	Ns	0.0, 0.0
Bare ground thaw/mud day	5	0	+1.5	0.7	+1.4, +2.6	1	1	0.0	ns	−1.9, +2.0	9	0	+2.0	1.6	+1.3, +2.3

*Notes*

Median (med slope) and range (range of slopes) of trends over time (d/decade) were calculated from Sen slopes in sites where trends were significant (α = 0.05), and No. pos and No. neg indicate number of significant positive and negative trends, respectively. Regional trends (reg slope) were determined using Sen slope analyses; ns indicates lack of significance. Statistics are reported for each of three subregions.

**Table 3:** Summary of long‐term median and range of indicators (number of days) relevant to forest ecosystems and surrounding communities

Indicator name	West		Central		East	
	Median	Range	Median	Range	Median	Range
Thaw day	121	91–156	164	145–173	142	121–194
Ice day	85	48–115	40	34–61	65	16–83
Frost day	164	142–174	123	116–149	138	71–164
Extreme cold/pine beetle kill day	41	18–70	5	3–10	19	0–45
Hemlock Woolly Adelgid Kill Day	5	0–19	0	0 ‐0	0	0–5
Snowmaking/mosquito kill day (before 28 February)	92	74–103	54	49–70	74	26–91
Snowmaking/mosquito kill day (before 25 December)	100	73–114	55	48–74	76	23–100
Snow covered day	130	77–153	61	52–95	100	32‐ 142
Bare ground day	80	59–134	150	116–161	112	68–182
Rain‐on‐snow day	1	0–4	1	0–3	4	0–11
Bare ground ice/frozen ground day	2	1–7	4	2–7	3	1–6
Bare ground thaw/mud day	76	56–124	142	112–153	106	67–173

*Note*

Summary statistics are calculated by site within each of three subregions.

## Results

### Coldness

**Figure 2:**
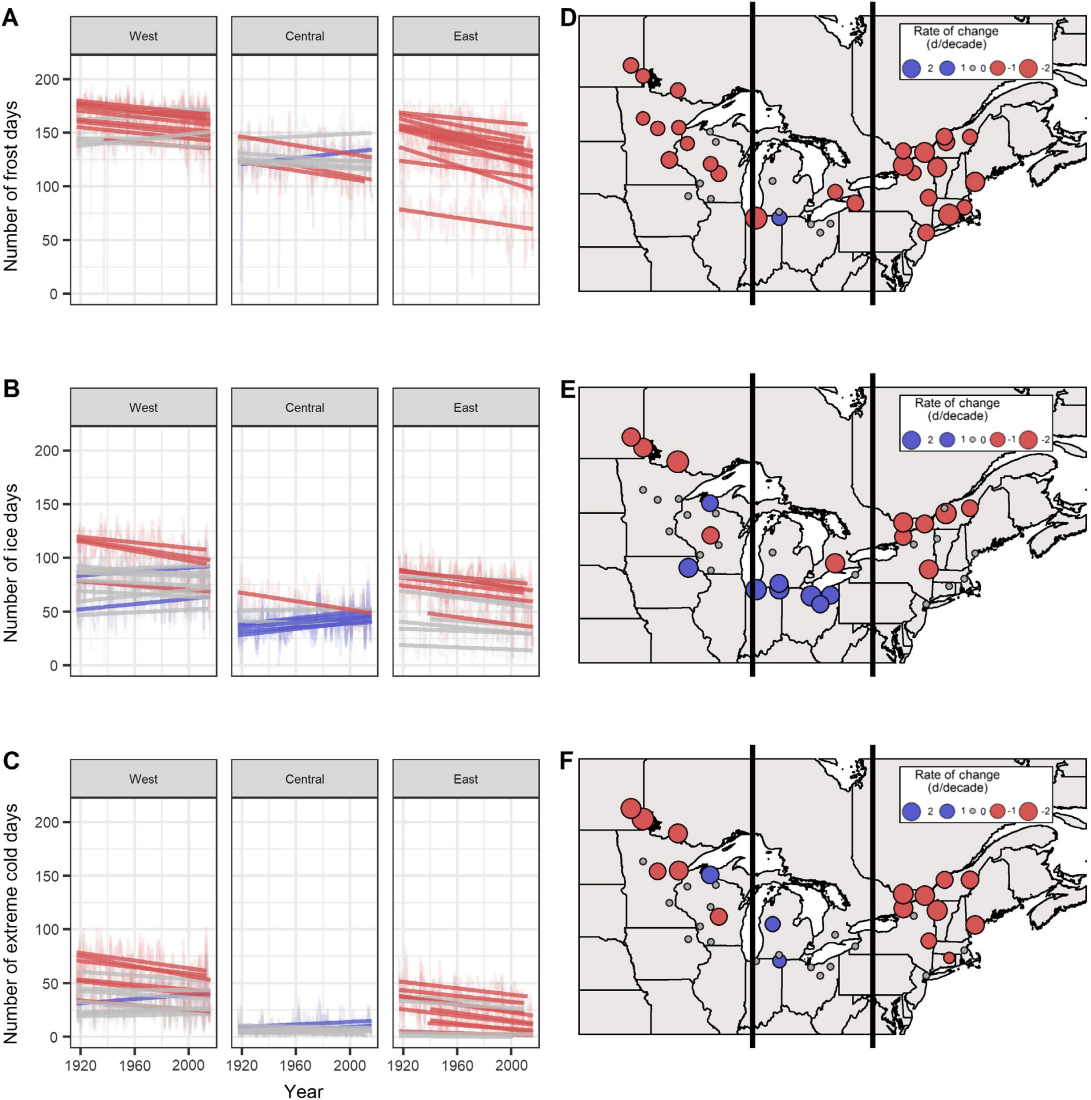
Change over time of general indicators of winter coldness. Panels A, B, and C show change over time for the number of Ice Days, Frost Days, and Extreme Cold Days, respectively, for each site over a 100‐year period from 1917 to 2016. Lighter‐colored lines in the background are time series for each site showing number of days per year that (A) Frost Days, (B) Ice Days, or (C) Extreme Cold Days occurred, while darker‐colored, straight lines in the foreground indicate trends. Red lines show decreasing trends, that is, reduced frequency of Ice, Frost, and Extreme Cold Days, over the time series. Blue lines indicate increasing trends, while gray lines indicate a lack of significant change over time. The intensity of the color corresponds to the significance of the trend. Sites are grouped into three geographic subregions, west, central, and east, to facilitate data visualization and interpretation. Panels D, E, and F display rates of change for Frost Days, Ice Days, and Extreme Cold Days, respectively, over the entire study area. Colors are the same as in panels A, B, and C, with red dots showing negative trends, blue dots showing positive trends, and gray dots showing no significant change. The size of the dot illustrates the magnitude of change.

The frequency of Frost, Ice, and Extreme Cold Days generally decreased over time, while the number of Thaw Days increased, with the magnitude of these trends varying spatially (Table 2, Fig. 2). We found overall declining trends in all indicators that quantified “coldness” (Ice Day, Frost Day, Extreme Cold Day) in western and eastern subregions, and declines in only Frost Days in the central subregion. Ice Days (*T*_max_ < 0°C) had the least consistent trends across the whole study area as compared to other indicators of coldness. Only 27% of western sites, 11% of central sites, and 38% of eastern sites showed significant declines in Ice Days over the past 100 years. In fact, Ice Days increased in 67% of central sites. By contrast, Frost Days displayed the most coherent pattern of all general coldness indicators, with all western and eastern sites and three of four central sites having a statistically significant decline in the number of days when daily minimum temperature was <0°C. This change amounted to a regional decrease of 1.1 frost days per decade (median slope = −1.4, minimum slope = −3.8, maximum slope = +1.2 d/decade) across the entire northern forest (Appendix S1; Table S3). Regarding Extreme Cold Days (*T*_min_ < −18°C), the overall trend was similar to that of Ice Days; central sites exhibited few significant trends over time, even as 40% of western sites and 70% of eastern sites showed declining trends for this indicator. Although there was a regional decline of 0.3 Extreme Cold Days per decade (median slope = 1.2, minimum slope = −2.2, maximum slope = +1.2 d/decade) across the entire northern forest domain (Appendix S1; Table S3), these events were rare in the southern parts of all subregions, particularly within the central sites, where the long‐term medians ranged from 3 to 10 Extreme Cold Days per year (Table 3).

**Figure 3:**
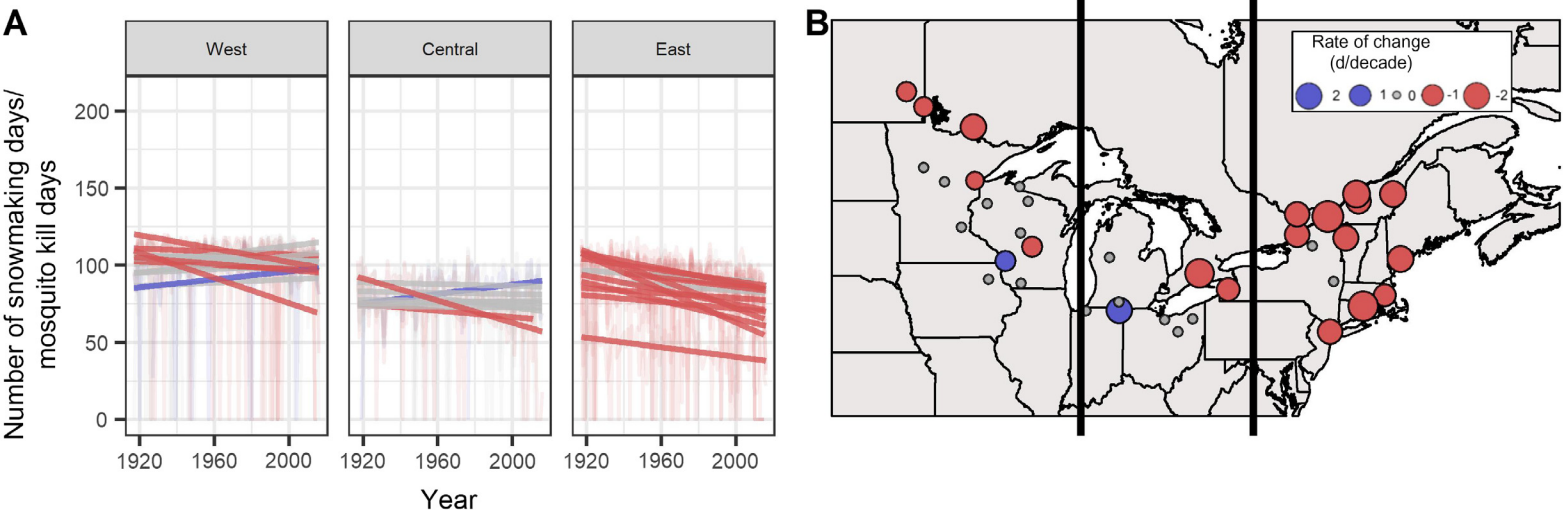
Change over time for number of days when conditions are suitable for snowmaking using a threshold of daily minimum air temperatures <−5°C (which is also the criteria for killing mosquitoes). Panel A shows change over time for Snowmaking Days for each site over a 100‐year period from 1917 to 2016. Panel B displays rates of change (as days per decade) in the frequency of Snowmaking Days over the entire study area. Lines, points, and colors are as in Fig. 2.

We observed reductions in Hemlock Woolly Adelgid Kill Days, Pine Beetle Kill Days, Mosquito Kills Days, and Snowmaking Days, all exemplifying potential effects of winter climate change on forest ecosystems and surrounding communities (Table 2). Hemlock Woolly Adelgid Kill Days, in which *T*_min_ < −30°C, declined by a median 0.3 d/decade (minimum slope = −1.1, maximum slope = 0 days per decade; Appendix S1; Table S2). These declines all occurred in the western and eastern subregions of the northern forest (Table 2), primarily in the northern parts of both subregions. Pine Beetle Kill Days were equivalent in our analysis to Extreme Cold Days (*T*_min_ < −18°C), and thus exhibited the same patterns described previously. Mosquito Kill Days were defined as days when daily minimum temperature was <−5°C, which was the same threshold applied to define Snowmaking Days. Fig. 3 shows the number of Snowmaking Days available before the Christmas holiday in December. Similar to other temperature indicators, the trends in Snowmaking Days prior to Christmas were strongest and most consistent in the east, where 11 of 13 sites show significant decreases, and the rates of change ranged from −0.9 to −2.3 fewer Snowmaking Days before Christmas per decade (Table 2).

### Snowpack

**Figure 4:**
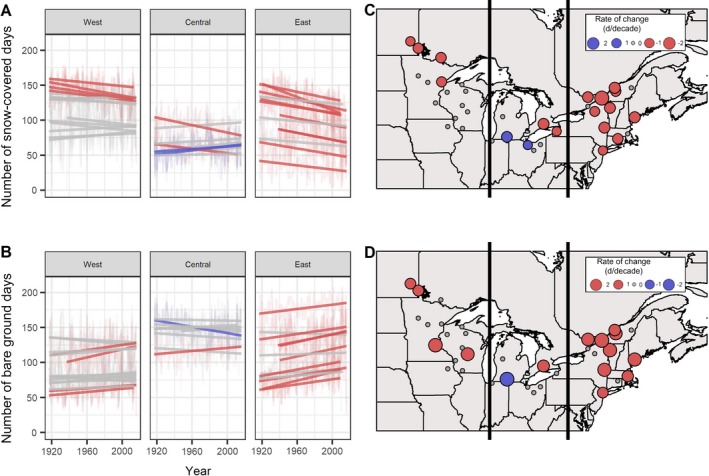
Change over time of indicators of winter snow cover. Panels A and B show change over time for snow covered days and bare ground days, respectively, for each site over a 100‐yr period from 1917 to 2016. Panels C and D display rates of change for snow covered days and bare ground days, respectively, over the entire study area. Lines, points, and colors are as in Fig. 2.

Changes in Snow Covered Days (snow depth > 0 mm) and Bare Ground Days (snow depth = 0 mm) were less consistent across the study region compared to temperature‐derived indicators (Table 2, Fig. 4). In the west and east, all statistically significant trends in Snow Covered Days were negative and represented 27% and 77% of sites, respectively. In these subregions, Snow Covered Days declined by an overall 0.8 d/decade in the west (median slope = −1.7, minimum slope = −2.2, maximum slope = −0.9 d/decade; Table 2) and by an overall 1.9 d/decade in the east (median slope = −2.1, minimum slope = −4.5, maximum slope = −1.2 d/decade; Table 2). In contrast, 22% of central sites showed significant increases in the number of Snow Covered Days, gaining as much as +2.0 d/decade (minimum slope = −2.1, median slope = −0.1d/decade, Table 2). Trends in the frequency of Bare Ground Days were opposite those of Snow Covered Days, typically increasing in tandem with the loss of snow cover.

Rain‐on‐Snow Days (liquid precipitation > 0 mm and snow depth > 0 mm) exhibited few changes in frequency per year, and the direction of the trend was mixed. We detected significant trends at only four sites in each of the east and west subregions (mixed trend direction), and two sites in the central subregion (both increasing). However, there were relatively few Rain‐on‐Snow Days across the 100 year time series, with zero or one event per year in many sites, and a maximum of 11 events within a single year at one site (Table 3).

### Coldness plus snowpack

**Figure 5:**
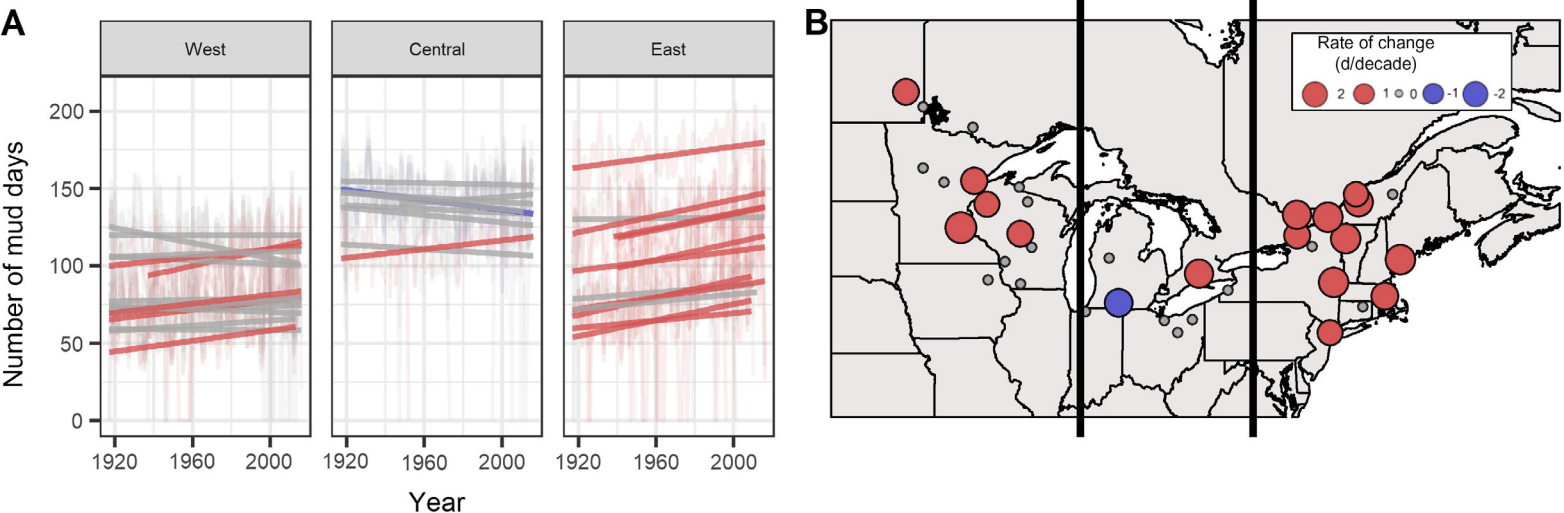
Change over time for number of Mud Days when snow cover is absent and daily maximum temperatures are >0°C. Panel A shows change over time for Mud Days for each site over a 100‐yr period from 1917 to 2016. Panel B displays rates of change in the frequency of Mud Days over the entire study area. Lines, points, and colors are as in Fig. 2.

We developed two indicators that reflected both temperature and snowpack characteristics together: Bare Ground Ice Days (i.e., Frozen Ground Days) and Bare Ground Thaw Days (i.e., Mud Days; Table 2, Fig. 5). These indicators represented potential impacts of changing winter conditions on ecosystem function, timber harvesting, and recreation and tourism. Only three of 37 sites across the entire study region showed significant increases in potential Frozen Ground Days (*T*_max_ < 0°C and snow depth = 0 mm), two central sites and one western site. As with Rain‐on‐Snow Days, these Frozen Ground Days were relatively infrequent, occurring between one and seven times per year (Table 3). By contrast, the frequency of Mud Days (*T*_max_ > 0 °C and snow depth = 0 mm) was sufficient to identify trends in the 100 year data set (Fig. 5). We note that inclusion of the “shoulder seasons,” or days in November and May that do not feature typical “winter” conditions, predetermined that there would be a relatively large number of days without snow that were above freezing in the data set. In the western and eastern subregions, all significant trends (at 33% and 77% of sites, respectively) were positive, ranging from +1.4 to +2.6 days without snow and above freezing per decade increase in these areas (Table 2). Again, the central subregion had mixed trends centered close to zero, indicating few changes over time (Table 2).

## Discussion

Winter temperatures (Thaw, Ice, and Extreme Cold Days) and snow cover (Snow Covered Days, Bare Ground Days) have changed across the northern forest region, with cold and snow covered conditions decreasing over the past 100 years. Few studies have assessed a comparable suite of general winter temperature and snowpack indicators over large spatial (regional to continental) or long temporal (>30–60 yr) scales, particularly with data that span both the northern United States and Canada. Studies that have examined winter climate change since about 1950 at global to regional scales have shown increases in winter (assessed as December‐February) temperatures, particularly nighttime temperatures, that exceeded increases in temperatures in other seasons (Vincent and Mekis 2006, Hayhoe et al. 2007, Donat et al. 2013, Vincent et al. 2015). Prior research has also demonstrated decreases in snow cover duration since about 1950, both in the northeastern United States and Canada (Burakowski et al. 2008, Vincent et al. 2015). Our findings support this general pattern of warming winter temperatures and reduced snow cover, but add further insight into potential societal and ecological impacts of such changes through the analysis of a unique suite of winter climate change indicators. The overall trend we observed toward increasing numbers of Thaw Days and Bare Ground Days and decreasing numbers of Frost Days and Snow Covered Days suggest changes to ecohydrology, soil microclimate and soil biological process, fine root dynamics, predator–prey dynamics, herbivory, and other ecological dynamics in a northern forest that is historically adapted to cold, snowy conditions. Some of the potential effects of changing winter across the region may even counterbalance one another. For example, lower numbers of days with coldness and/or snow, whether Frost Days, Extreme Cold Days, Snowmaking Days, Snow Covered Days or Frozen Ground Days, suggest positive outcomes for tree health as related to reduced fine root mortality and nutrient loss associated with winter frost (Cleavitt et al. 2008, Fuss et al. 2016). At the same time, the overall loss of coldness and snow cover might have negative consequences for tree health as related to the northward advancement and proliferation of forest insect pests. Declines in coldness and snow cover may also carry negative consequences for logging and forest products, vector‐borne diseases and human health, recreation and tourism, and cultural practices, which together represent important social and economic dimensions of the provinces, states, Tribal Nations, First Nations, Indigenous communities, cities, and communities of the northern forest region.

### Changes in general winter climate conditions

#### Coldness

We found overall declining trends in number of days that quantified general “coldness” (Ice Days, Frost Days, and Extreme Cold Days), with Frost Days exhibiting the most coherent, decreasing trends across the study region. Other studies have documented similar responses, with 57%–65% of stations in the northeastern United States (1893–2005 [Brown et al. 2010], 1926–2000 [Griffiths and Bradley 2007]) and 62% of sites across Canada (1900–2003 [Vincent and Mekis 2006]) exhibiting decreases in Frost Days, and ~97% of sites globally declining in “cool nights” (percentage of time when daily minimum temperature <10th percentile”, 1901–2010 [Donat et al. 2013]). Prior investigations have also reported similar rates of change over time. Across the northern forest, Frost Days declined by −0.8 to −3.8 days per decade. This trend is consistent with the findings of Brown et al. (2010), who reported a decline in Frost Days at a rate of −2.1 d/decade across the northeastern United States from 1951 to 2005. It also fits with Anandhi et al. (2013), who noted a decline of −3.1 to −6.6 d/decade in the number of Frost Days from 1960 to 2000 for five watersheds in the Catskills region of New York.

For Ice Days (*T*_max_ < 0°C), we found fewer significant trends, weaker magnitudes of change, and greater differences among study subregions compared to other indicators. A smaller number of studies have reported trends in Ice Days compared to trends in Frost Days; however, in the northeastern United States, Brown et al. (2010) noted that for all colder minimum and maximum temperature indices (including Ice Days), more than one‐half of the stations had significantly declining trends from 1870 to 2005. In stations across the globe from 1951 to 2010, 39.4% of sites had a significant decrease in number of Ice Days, while 3.1% had a significant increase in number of Ice Days (Donat et al. 2013). These statistics align with our finding that Ice Days generally decreased in the west and east while increasing in the central subregion.

Due to lack of an analogous indicator, we are unable to directly compare Extreme Cold Days/Hemlock Woolly Adelgid Kill Days (*T*_min_ < −18°C) to values previously reported. However, temperature records for 22 sites in the northeastern United States for the period 1951–1993 showed significantly decreasing trends in the number of days with minimum temperatures ≤−15°C (DeGaetano 1996), and this agrees with our finding that Extreme Cold Days significantly declined in both the West and the Northeast over the past 100 yr.

For the central subregion, the positive or mixed trends we detected for temperature indicators such as Ice Days and Extreme Cold Days fits with previous findings of both decreasing or unchanging winter temperatures in the central United States (Andresen et al. 2012, Mascioli et al. 2017). Long‐term phase changes in the ocean–atmosphere circulation modes such as North Atlantic Oscillation may explain an overall cooling or mixed temperature trend in this area, though the causes of the United States “warming hole” are highly uncertain (Mascioli et al. 2017, Partridge et al. 2018). In addition, we note that the weather stations we used to identify trends in this subregion were located toward the southern portion of the study area. Data were absent for the Upper Peninsula of Michigan and for central and eastern Ontario, limiting the inferences we can draw about general changes in winter coldness for this portion of the central subregion.

#### Snowpack

Our result of declining Snow Covered Days (snow depth > 0 mm) fits with previous studies in both North America and across the northern hemisphere showing that the snowpack is thinner, the snow season is shorter, and snow cover is less continuous (Dyer and Mote 2006, Kreyling 2013, Vincent et al. 2015). This was particularly evident in the east and west subregions, which exhibited median loss rates from −1.2 to −4.5 d/decade that were comparable to the average loss of −3.6 d/decade calculated by Burakowski et al. (2008) for the northeastern United States over the period of 1965 to 2005. Though not a perfect analog, Durán et al. (2016) similarly reported that time‐integrated snowpack depth (depth × duration) decreased significantly from 1971 to 2012. Likewise, Vincent et al. (2015) noted that snow cover duration (as defined by number of days when snow depth ≥2 cm) declined by −1.0 to −3.4 d/decade during 1950 to 2012. The central subregion had the fewest Snow Covered Days of all three subregions in our study, with a median of only 60.5 Snow Covered Days over the 100‐yr time series (Table 3). Increases and mixed trends in related indicators such as snowfall and length of snow season in the Great Lakes area, where central sites were primarily located, have previously been reported for the 20th century, driven by lake‐effect snowfalls, particularly since 1970 (Brown 2000, Kunkel et al. 2009). The combination of warming water, reduced ice cover, and increased evaporation may drive these increasing snowfall trends in the Great Lakes (Kunkel et al. 2009), though future winter warming may ultimately lead to increased rainfall over this subregion (Notaro et al. 2015).

### Potential impacts on ecosystems of the northern forest

#### Water

Changing winter temperature, precipitation, and snowpack conditions can impact ecohydrology by altering stream water quality and quantity and the timing of lake ice formation and loss. Regarding stream water quantity, prior studies examining changes in winter air temperatures have reported that warmer conditions, particularly from February through May, result in both more frequent mid‐winter melt events and earlier spring snow melt (Hodgkins et al. 2003, Dudley et al. 2017). While we did not examine linkages among changing air temperatures, snowmelt, and streamflow, the trend we observed toward increasing frequency of Thaw Days, particularly in the west and the east, suggests that the spring freshet may also occur earlier in these subregions and be smaller in magnitude as compared to historical spring snow melt. Such shifts may carry consequences for both forest ecosystem water balance (Hodgkins and Dudley 2006, Creed et al. 2015), and in populated areas may also severely impact water resources (Barnett et al. 2005).

In addition to effects on water quantity, changing winter conditions may also alter water quality. Recent studies (Huntington and Billmire 2014, Huntington et al. 2016) have shown that trends in increased winter temperatures, more frequent winter rains, and greater overall winter precipitation have increased winter runoff that, in turn, may increase the rate of leaching of base cations and dissolved organic carbon (Huntington 2005, Rustad et al. 2012). Rain falling on frozen ground or saturated soils may further increase rates of winter runoff (Shanley and Chalmers 1999, McMillan et al. 2018), though we were unable to examine such phenomena with our data set. In addition to these general trends, an increase in the frequency of rain‐on‐snow events could have implications for water quality. While rain‐on‐snow may have been a rare occurrence in the past, it is likely to become more common in the future (Leung et al. 2004, Ye et al. 2008, Casson et al. 2010, 2012), and indeed has already increased in Arctic regions by up to 50% (Williams et al. 2015). While we observed few significant trends toward increased frequency of Rain‐on‐Snow Days, the apparent rarity of these events over our 100‐year time series may have precluded strong inference about changes in their occurrence. Further, the trend we observed of fewer Snow Covered Days may have led to fewer days with snow cover on which rain could fall; Mukundan et al. (2013) suggested this synergy could mask trends in simulated rain‐on‐snow events in the Esopus Creek watershed that acts as a water supply for New York City, USA. Despite their apparent rarity, existing literature demonstrates that when these events do happen, they have a disproportionately large effect on hydrochemistry, including accounting for 12–42% of annual nitrate export (Casson et al. 2012, Kurian et al. 2013, Crossman et al. 2016), and potential acid pulses (Eimers et al. 2007).

As for impacts on lakes, prior studies have extensively documented the effects of winter climate change on lake ice. Across both North America and the globe, lake ice‐out dates have been advancing as a result of warmer air temperatures, resulting in a dramatic shortening of the ice‐covered season (Magnuson et al. 2000, Hodgkins et al. 2002, Sharma et al. 2016). Like changes in the timing of snowmelt, our findings of decreased numbers of Frost Days and increased numbers of Thaw Days, particularly in the western and eastern subregions, fit with these prior studies and indicate that warmer air temperatures, particularly above 0°C, may advance lake ice‐out dates throughout our study domain. The loss of lake ice carries consequences for lake ecology (Hampton et al. 2017), fish populations (Brander 2007), nutrient cycling (Powers et al. 2017), and greenhouse gas emissions (Denfeld et al. 2018). Beyond these ecological impacts, frozen lakes are important for winter recreation, including snowmobiling, ice fishing, and hockey, and loss of consistent frozen conditions may negatively impact these activities and the rural economies they support (McBoyle et al. 2007, Scott et al. 2008).

#### Soil

Because the snowpack insulates soil from freezing, many previous studies have considered how a combination of reduced snow cover plus cold temperatures might increase soil frost, which, in turn, affects microbial biomass, fine roots, and soil aggregates, and thus soil nutrient and carbon retention (Fitzhugh et al. 2001, Hardy et al. 2001, Groffman et al. 2001, 2006, 2011, Neilsen et al. 2001, Decker et al. 2003, Cleavitt et al. 2008, Campbell et al. 2014, Comerford et al. 2013, Durán et al. 2014, Fuss et al. 2016, Patel et al. 2018). Our results indicate that the frequency of potential soil freezing days (i.e., Bare Ground Ice Days/Frozen Ground Days when *T*_max_ < 0°C and snow depth = 0 mm) that might affect soil physical, biological, and biogeochemical processes and properties did not significantly change over our 100‐year time series, and in fact, were somewhat rare occurrences (Table 3). While our results fit with those of other studies (Campbell et al. 2010, Brown and DeGaetano 2011), they should be interpreted with caution as they are based on the presence or absence of modeled snow depth and air temperature data. A modeled snowpack that we counted as a Snow Covered Day may have been too shallow to insulate soils from cold temperatures that could result in soil frost (Brooks et al. 2011), and thus, we may have underestimated long‐term trends in soil freezing. However, a lack of high‐quality snow depth and soil temperature data across the region, particularly over multi‐decadal timescales, precluded our ability to analyze relationships among snow depth, air temperature, and soil temperature, highlighting the need for such a regional network of observations. Further, our analyses were limited to soil freezing and not soil freeze/thaw cycles, which have already increased and are projected to increase over the next century (Hayhoe et al. 2007, Campbell et al. 2010, Brown and DeGaetano 2011).

Unlike Frozen Ground Days, the frequency of Bare Ground Thaw Days/Mud Days (*T*_max_ > 0°C and snow depth = 0 mm) significantly increased in both the west and east subregions of our study domain. These Mud Days can occur within the core of winter (December–February) as mid‐winter thaws, which prior studies have suggested will become more common in the future (Campbell et al. 2010, Sinha and Charkauer 2010). When coupled with increasing trends of winter rainfall, such mid‐winter Mud Days can result in saturated soils. If refrozen, concrete frost formation can occur (Fahey and Lang 1975, Proulx and Stein 1997, Tatariw et al. 2017), altering soil carbon and nitrogen availability (Patel et al. 2018), reducing groundwater permeability, and increasing surface water runoff (Shanley and Chalmers 1999). Mud Days may also become more frequent during the months of March and April as the ecosystem transitions between the growing and dormant seasons and the timing of snowmelt advances earlier in the year. The ecological effects of more numerous Mud Days during the vernal transition are not well understood. Prior studies examining mid‐winter thaws have generally coupled such events with subsequent freezing to determine the impacts of freeze–thaw cycles on soil physical properties, microbial biomass, greenhouse gas emissions, and/or nutrient cycling (Schimel and Clein 1996, Groffman et al. 2001, Edwards et al. 2007, Schimel et al. 2007, Aanderud et al. 2013, Tatariw et al. 2017, Patel et al. 2018). While numerous studies have reported both earlier snowmelt (Dyer and Mote 2006, Vincent et al. 2015) and canopy leaf out (Schwartz et al. 2006, Post et al. 2018) as a result of climate change, the intervening period of relatively warm, snow‐free soils, that is, the vernal window when Mud Days might occur, has received much less attention and is an important area for future research (Contosta et al. 2017). For example, the increases in soil respiration and soil carbon loss that occur during the growing season as a result of warmer soil temperatures (Rustad et al. 2012) may also occur on Mud Days during the vernal transition.

#### Vegetation

Prior work examining the direct response of vegetation to winter conditions has largely quantified the impacts of (1) extremely cold air temperatures on foliar tissues of red spruce (*Picea rubens* Sargent; Hawley et al. 2006, Lazarus et al. 2006, Kosiba et al. 2013, Schaberg et al. 2011), (2) freeze–thaw cycles on the xylem and root tissues of both yellow birch (*Betula alleghaniensis* Britton; Zhu et al. 2002, Bourque et al. 2005) and paper birch (*Betula papyrifera* Marshall; Cox and Malcolm 1997) and (3) soil frost on fine roots (Tierney et al. 2001, Cleavitt et al. 2008, Auclair et al. 2010, Comerford et al. 2013, Campbell et al. 2014, Reinmann and Templer 2016), foliage (Comerford et al. 2013), and aboveground growth (Reinmann and Templer 2016; Reinmann et al. 2019) of a variety of hardwood species.

Our results indicate that the Extreme Cold Days (*T*_min_ < −18°C) that would be required to induce red spruce injury have decreased in frequency over the past 100 years. While we did not explicitly examine the freeze‐thaw conditions that would affect the xylem and root tissues of yellow and paper birch, the trend toward more frequent Thaw Days and less frequent Ice and Frost Days across the region suggest reduced risk for winter dehardening followed by freezing injury and dieback. Likewise, the conditions necessary to induce soil frost and fine root mortality across a variety of species, that is, cold temperatures in the absence of snow cover during bare ground ice days or soil frost days, have also become less frequent over the past century. However, projections for increasing soil freeze/thaw cycles suggest potential damage to roots of maple species, affecting their ability to take up and retain nutrients such as nitrogen (Templer et al. 2017, Sanders‐DeMott et al. 2018a). In addition to the changes in winter conditions that could impact vegetation health, longer and warmer growing seasons, as indicated by increased numbers of Thaw Days, could exacerbate or offset these effects (Templer et al. 2017). Numerous other studies have reported earlier growing season onset due to warmer air temperatures (Richardson et al. 2006, Schwartz et al. 2013, Paio et al., 2015) that ultimately may lead to increased forest productivity (Richardson et al. 2009).

#### Forest insect pests

The northern forest features a large and growing number of active and potential forest pests (Dukes et al. 2009, Weed et al. 2013, Lovett et al. 2016, Ayres and Lombardero 2018). Many of these insects experience mortality from lethal winter cold, though the importance of winter temperatures for insect abundance is variable among species and geographic regions (Bale and Hayward 2010, Weed et al. 2015). In some cases, changing winter conditions can affect the distribution and abundance of forest pests by reducing their exposure to lethally cold temperatures. Relaxation of previous constraints from winter cold may add to the number of species for which the region is climatically suitable. We used long‐term meteorological data to calculate indicators that represent relaxation of these constraints. The indicators were based on two insects whose potential for current and future impacts are clearly related to the occurrence of lethally cold winter temperatures.

The southern pine beetle is native to North America, especially forests of loblolly pine (*Pinus taeda* L.) and shortleaf pine (*Pinus echinata* Miller) in the southeastern United States. It is one of the most aggressive tree‐killing insects in the world, impacting both the forest products industry and forest ecosystems in general (Coulson and Klepzig 2011, Pye et al. 2011). About one‐half of the beetles die from exposure to a single winter night when the air temperature drops to −17°C, and mortality is >90% if the coldest night drops to −22°C (Ungerer et al. 1999, Lombardero et al. 2000, Trân et al. 2007). The warming of the coldest night of the winter (>4°C in 50 years) has facilitated the northern expansion of the beetle about 200 km beyond its historic range into the pinelands of New Jersey, New York, and Connecticut (Dodds et al. 2018). Climate projections for the next 50 years indicate continued warming of the coldest night of the winter will be sufficient to permit further expansion of southern pine beetle into much of the northeastern United States and southeastern Canada (Lesk et al. 2017).

The hemlock woolly adelgid (*Adelges tsugae* Annand) is an invasive sapsucking insect from Japan that feeds in the crown of hemlocks and causes declining vigor followed by death of the host tree a few years after initial infestation (Dukes et al. 2009). Since its introduction to North America in the early 1950s, the adelgid has caused widespread mortality of eastern hemlock (*Tsuga canadensis* L.) in infestations from northern Georgia to the southern counties of New York, Maine, New Hampshire, and Vermont (Evans and Gregoire 2007). The loss of hemlock from hemlock wooly adelgid has been both direct (trees dying from adelgid attacks) and indirect (preemptive cutting of hemlock by landowners and forest managers in anticipation of its arrival; Orwig et al. 2002). Populations of hemlock woolly adelgid begin to experience mortality when winter air temperatures decline below −20°C and mortality is nearly complete if temperatures reach −30°C (Skinner et al. 2003, Tobin et al. 2017). Similar to southern pine beetles, previous limits on the northern distribution of the adelgid are being relaxed by amelioration of winter cold (Fitzpatrick et al. 2012).

Our indicators of Pine Beetle Kill Days and Hemlock Woolly Adelgid Kill Days were based on a simple temperature threshold but nonetheless captured winter warming trends consistent with actual range expansions of both pests (Table 2). This is not only relevant to anticipating future distributions of these particular insects, but may also signal relaxed distribution limits for numerous other plant and animal species. Our analyses add to the spatial extent of knowledge regarding trends in minimum annual air temperature. Previous work has shown similar warming patterns in eastern and western North America of 2°–4°C over 50 years (Trân et al. 2007, Weed et al. 2015). Trends in the reduction of Hemlock Woolly Adelgid in the western and eastern subregions fit with these prior analyses. In central sites, the lack of loss of kill days was likely due to the fact that this geographic area had few of these days to lose over the 100‐year time series (Table 3).

Another forest pest that is of great concern to natural resource managers, foresters, municipalities, Tribal Nations, First Nations, and Indigenous communities across this region is the emerald ash borer (*Agrilus planipennis* Fairmaire), an invasive Eurasian beetle whose larvae bore through the outer bark of otherwise healthy ash trees to feed in the phloem and cambium, essentially girdling the trees’ trunks and branches (Herms and McCullough 2014, Lovett et al. 2016). The prepupae accumulate high concentrations of glycerol and other antifreeze agents in their body fluids, making them extremely cold tolerant, with kill temperatures as low as −35.3°C (Crosthwaite et al. 2011). As such, we did not calculate a separate indicator for the emerald ash borer, whose distribution is ultimately more limited by host tree availability than climatic factors (Sobek‐Swant et al. 2012).

#### Wildlife

Changing winter weather can impact wildlife by altering snow depth, snow physical characteristics, or timing of snow cover, by shifting air temperatures, or through a combination of changes in temperatures and snowpack. The effects of changing winters on wildlife populations are further complicated by interactions among climate variables, land use, forest type, forest harvest, and the impact of all these on predation dynamics (Patterson and Power 2002, Visscher et al. 2006, Diefenbach et al. 2016). For example, the effect of snow depth on browse varies both between forest types and with animal size, such that the reductions we observed in number of Snow Covered Days might be beneficial from the standpoint of larger mammals occupying coniferous forests where even a 20‐cm snowpack can severely reduce available browse vegetation (Visscher et al. 2006). At the same time, reduced frequency of Snow Covered Days might be detrimental to smaller mammals who gain access to browse higher up on shrubs when snowpacks are deep (Nordengren et al. 2003). Further, shifts in wildlife browsing activity that result from changes in snow depth could have cascading effects on regeneration of forest plants in the understory (Christenson et al. 2014, Sanders‐DeMott et al. 2018b). Because snow cover provides subnivean refuge, the reductions we observed in Snow Covered Days may also translate into limits on small mammal abundance as well as enhanced competition among predators, for example allowing red fox (*Vulpes vulpes* L.) to outcompete Arctic fox (*Vulpes lagopus* L.; Penczykowski et al. 2017).

Regarding snow physical characteristics, increased freezing and thawing, or simply warmer winter temperatures and more frequent Thaw Days, will likely also alter depth, density, and hardness of remaining snowpack and connectivity of the subnivean space, which may influence access to food and refuge for small mammals from their predators (Bilodeau et al. 2013). Likewise, reduced snow depth or formation of ice crusts in warmer, wetter winters could impede the ability of ruffed grouse (*Bonasa umbellus* L.) to snow roost, or bury themselves in insulating snow, leading to increased thermoregulatory costs (Thompson and Fryzel 1988), reduced survival (Zimmerman et al. 2008), and ultimately population decline. Changes in the length of snow covered season, as evidenced by decreasing numbers of Snow Covered Days and increasing numbers of Bare Ground Days, can alter the visual refuge of the snowpack by creating camouflage mismatch for snowshoe hare (*Lepus americanus* Erxleben; Zimova et al. 2014, 2016) and its predators, including is Canada lynx (*Lynx Canadensis* Kerr), coyote (*Canis latrans* Say), Goshawk (*Accipiter gentilis* L.), and great horned owl (*Bubo virginianus* Gmelin; Feierabend and Kielland 2015).

An overall warming trend in winter temperatures, as illustrated by increased frequency of Thaw Days across the western and eastern subregions, may also negatively impact wildlife by exposing them to disease. Moose (*Alces alces* L.), strongly associated with natural heritage and tourism in the northern forest, have been affected by parasites such as winter tick (*Dermacentor albipictus* Packard) that can flourish during warmer winters (Dunfey‐Ball 2017). At the same time, survival of white‐tailed deer (*Odocoileus virginianus* Zimmermann) can be strongly influenced by winter severity (which encompasses both snow depth and temperature), with losses ranging from negligible to ~30% depending on winter conditions (Lavigne 1999).

For wildlife that is migratory or endemic to high elevations, winter climate change effects are difficult to isolate from changes in the “shoulder” seasons or changes in the climate of the breeding habitat. Migratory birds may leave wintering grounds because of poor conditions only to return to breeding areas that are ahead or behind with respect to ideal temperature and food availability. Many of the neotropical migrant bird species that breed in our study area are able to adjust somewhat the timing of their spring return to the breeding grounds based on cues along the migratory route, but this is variable among species and the birds are generally less responsive in their migration phenology than local trees and insects are to local temperatures (Marra et al. 2005, Wilson 2007, Lany et al. 2016). With respect to high elevation species that live in spruce–fir habitat such as Bicknell's thrush (*Catharus bicknelli* Ridgway), warming winter temperatures may reduce its competitive advantage over the less cold‐tolerant Swainson's thrush (*Catharus ustulatus* Nuttall). In the short term, however, potentially more frequent ice storms (Cheng et al. 2011) could increase canopy disturbance in the higher elevation conifer forest and improve habitat features for this rare species (Lambert et al. 2005).

### Potential impacts on human communities of the northern forest

#### Human health

Winter weather can directly impact human health through exposure to cold temperatures that lead to frostbite and hypothermia, travel hazards due to the presence of ice and snow on roads and sidewalks, and winter storms and associated disruptions to power that can lead to accidents and exposure to both cold and carbon monoxide from generators. Winter conditions can also indirectly impact human health by allowing the spread of vector‐borne diseases whose ranges, like those of forest insect pests, can be at least partially limited by lower lethal temperatures (Beard et al. 2016). The decreases we observed in Ice, Frost, Extreme Cold, and Snow Covered Days suggest a declining trend in the direct human health impacts related to exposure to cold temperatures, winter storms, and dangerous travel conditions. Conversely, the trend toward fewer Mosquito Kill Days (i.e., when daily minimum temperatures are <−5°C) and more frequent Thaw Days (i.e., when daily maximum temperature >0°C) suggests the potential for increased exposure to insect vectors and other human health risks.

The emergence and spread of vector‐borne diseases is complex and can involve interactions among the introduction of invasive vector species, land use change, and alterations in human behavior (Allan et al. 2003, Kulkarni et al. 2015, Beard et al. 2016). However, numerous studies have demonstrated a linkage between milder winter temperatures and the spatial distribution of insect vectors such as the endemic blacklegged tick (*Ixodes scapularis* Say) that carries the *Borrelia burgdorferi* bacteria that causes Lyme disease (Brownstein et al. 2003, Leighton et al. 2012, Levi et al. 2015), and the invasive Asian tiger mosquito, an important vector for dengue and chikungunya fevers and eastern equine encephalitis (Platonov et al. 2008, Rochlin et al. 2013, Ogden et al. 2014). If our historical trends continue into the future, continued loss of cold conditions might contribute to the northward expansion of these and other insect vectors, with public health consequences (Kugeler et al. 2015). An increasing number of Thaw Days also leads to extended allergy seasons with enhanced pollen exposure, particularly for tree pollen in the northeastern United States. (Zhang et al. 2015). Shorter winters and rising atmospheric CO_2_ may increase the production and allergenicity of pollen, increasing risk of allergic disease to humans (Reid and Gamble 2009, Blando et al. 2012).

The human health risks associated with winter climate change can vary across individuals and groups of people, with people who spend more time outdoors and in tick habitat becoming more vulnerable to vector‐borne diseases than others. For example, a study in Poland demonstrated forestry workers performing manual jobs in the forest were statistically at greater risk for tick bites and tick‐borne diseases than administrative workers (Cisak et al. 2012). Also, cultural and subsistence practices of citizens of Tribal Nations, First Nations, and Indigenous communities can increase exposure to these risks. Tick‐borne illness transmission to Tribal citizens during activities such as hunting, fishing, and gathering plants/herbs are a major concern.

#### Winter logging and forest products

Winter forest harvesting often requires a sufficient snowpack or frozen soils, both to ensure easy access to sites on navigable roads, as well as to prevent environmental impacts such as soil compaction, erosion, and deposition of contaminants into surface waters, particularly on sites with medium to fine‐textured, poorly drained soils (Evans et al. 2016). Our results suggest a decline in conditions suitable for winter logging, namely a decrease in Snow Covered Days, a lack of change in Bare Ground Ice Days/Frozen Ground Days, and an increase in Bare Ground Thaw Days/Mud Days. Rittenhouse and Rissman (2015) also reported a loss of suitable conditions for winter logging from 1948 to 2012. They observed a two‐ to three‐week reduction in the period of frozen ground, which in turn, had implications for timing of logging. For example, species that grow in sandy, well‐drained areas, such as jack pine (*Pinus banksiana* Lamb.), were harvested at higher rates during years with greater soil thaw duration, while species, such as black spruce (*Picea mariana* Miller), which typically occur on moist sites or bottomlands, showed the opposite trend (Rittenhouse and Rissman 2015). Changes in winter logging conditions may also pose economic hardships, both to individuals engaged in the profession, as well as to surrounding communities. Forestry professionals may not be able to complete contracts on schedule if frozen ground is necessary to either facilitate site access and/or mitigate environmental impacts (Evans et al. 2016). Since forest resources remain a significant employment opportunity in many rural and Indigenous communities on forested landscapes, loss of work during the winter season may have also cascading impacts on local economies (Thill 2013, Mausel et al. 2017).

#### Recreation and tourism

The U.S. snow sports industry (Nordic and Alpine skiing, snowboarding, snowmobiling, and snowshoeing) supports ~695,000 jobs that contribute US$5.2 billion in state and local taxes (Outdoor Industry Association 2017). Likewise, Canadian skiing and snowboarding industries can generate significant revenues, estimated between US$600 million to US$1 billion (Canadian Ski Council 2018). Climate change has long been recognized as threatening these billion‐dollar contributions to local, provincial, and state economies, both within the northern forest and globally (Scott and McBoyle 2007). Within the northern forest region, downhill skier visitation is significantly correlated with snowfall (Hamilton et al. 2007, Dawson et al. 2013) and with the number of days with snow on the ground (Hagenstad et al. 2018), such that skier visitations decline during low‐snow winters (Scott and McBoyle 2007, Hagenstad et al. 2018).

Given the established relationships among winter weather, skier visitations, and ski area revenues, the multi‐billion dollar question is, “How will the ski industry respond to a warmer and less snowy climate?” Prior research has generally focused on the supply‐side of this question, exploring how ski areas might adapt to changing winters through business practices as well as with technological innovations (Scott and McBoyle 2007). Business practices such as revenue diversification can reduce ski area vulnerability to low snow or no‐snow winters. At the same time, technological innovations such as snowmaking have allowed ski resorts to increase the quantity and quality of snow despite a declining natural snowpack, increasing the overall length of the ski season and the acreage of trails (Scott and McBoyle 2007). Snowmaking has become increasingly energy and water‐efficient since its early beginnings as “shaved ice” in the 1930s (Hall 1934, Leich 2001). Likewise, technological advancements have enabled the creation of artificial snow at increasingly warmer temperatures, from a historical threshold of −5°C to a current threshold of −2°C (Scott et al. 2003, Wilson et al. 2018). Despite these improvements in snowmaking technology, we observed an overall decrease in the number of Snowmaking Days at both temperature thresholds and during the two “peak use” time windows: prior to 25 December and prior to 28 February (Appendix S1; Table S2). Rates of change in numbers of Snowmaking Days were generally greater prior to 25 December as compared to the entire snowmaking period, suggesting a delayed start to the ski season over time.

On the demand side, participants might respond to changing winters by altering the timing or location of snow sport activities. They might also pursue alternative recreation activities regardless of how much natural or artificial snow is present on the trails (Scott and McBoyle 2007). The presence of snow in urban locations is a well‐documented factor that drives skier demand independent of local conditions at resorts in the northeastern United States, known as the “backyard effect” (Hamilton et al. 2007). That is, skiers in both the northeastern United States and Canada have expressed a decreased willingness to travel (i.e., reduced spatial substitution) to more northerly, high‐elevation resorts if a snowpack is absent where they live (Dawson et al. 2013, Rutty et al. 2015). Snowmaking is thus a cautionary adaptation strategy, as it may not be enough to create the snow conditions needed for skiing to attract skiers to the slopes. In fact, significantly decreasing trends in Snow Covered Days and increasing trends in Bare Ground Days reported here, particularly at more southern locations in the northern forest, are consistent with previous findings that changes in winter climate have contributed to contraction and consolidation of downhill ski resorts in areas of the northeastern United States (Hamilton et al. 2003, Beaudin and Huang 2014). Continuation of these trends may lead to tens of millions of dollars in future economic losses from winter recreation for the northern forest region (Chin et al. 2018).

#### Indigenous peoples

The northern forest region is the original homeland of many Indigenous peoples, including citizens of federally and state‐recognized Tribal Nations in the United States, First Nations and the Métis Nation in Canada, and Indigenous communities throughout the region still seeking federal or state recognition. Tribal/First Nations and Indigenous peoples are intertwined with the area's forests in many important and substantial ways (Voggesser et al. 2013, Mausel et al. 2017). As a result, as discussed above, the winter climate changes we identify with this study and the associated ecological, social, and economic effects also relate directly to Tribal/First Nations and Indigenous peoples. Importantly, Norton‐Smith et al. (2016) also explain that the interconnectedness of Indigenous cultural practices, identities, and traditional knowledges with specific places, ecological processes, and species can compound the effects of climate change. Further exacerbating these effects are stressors related to colonialism, systemic racism, and forced relocation (Norton‐Smith et al. 2016, Whyte 2017).

Some of the cultural impacts of the winter climate changes we identify with this study have to do with specific species. For example, three species discussed above, birch trees, moose, and snowshoe hare, carry cultural and subsistence significance for Indigenous communities (Jacqmain et al. 2012, Norton‐Smith et al. 2016); Panci et al. 2018). Also, in the central area of our study, warming winters and hydrologic changes are shifting the range of wild rice (Minnesota Department of Natural Resources, 2008), a traditional food source that Lynn et al. (2013) describe as a “pillar of cultural health for the Anishnaabeg people” (Lynn et al. 2013:550).

The human health dimensions of winter climate change may also be compounded within Indigenous communities. Climate‐change‐related losses in access to and availability of traditional foods are tied to increases in modern diets, which in turn are linked to increases in rates of health problems such as type 2 diabetes and heart disease (Norton‐Smith et al. 2016). Also, the experience of ecological changes and the loss of access to lands, waters, plants, and animals of cultural significance can lead to depression, distress, and other mental health concerns among Indigenous peoples (Willox et al. 2015).

Despite these challenges, several scholars point to the demonstrated resilience of Tribal/First Nations and Indigenous peoples, rooted in multiple facets of traditional knowledge, social connections, diverse ways of living, and cultural values, as an important strength for addressing and adapting to climate change (Norton‐Smith et al. 2016, Whyte 2017). Indeed, Tribal/First Nations are at the forefront of many climate‐change adaptation and mitigation efforts (Whyte 2017).

## Conclusion

Northern forest winters are losing the cold, snowy conditions upon which ecosystems and people rely. Declines in the numbers of Frost Days, Snow Covered Days, and other indicators of winter cold and snow cover may negatively impact social and economic activity related to winter based forestry activities and tourism while also increasing the incidences of vector‐borne diseases and pollen‐related allergies. The increases we observed in Thaw Days and Bare Ground Days may result in the northward advancement of forest insect pests, declining water quantity and quality, and increased stress to small and large mammals, birds, and fish alike while at the same time reducing winter injury to tree species such as red spruce and winter mortality to mammals such as white‐tailed deer. While winter climate change may inspire industry adaptations, these innovations may only go so far in supporting industries that have historically relied upon cold, snowy conditions. The loss of cold and snow may require fundamental changes of the socioecological system of northern forests.

## Data Availability

Associated code and data are available on Zenodo: https://doi.org/10.5281/zenodo.3248905

## Notes

1 https://www.ncdc.noaa.gov/cdo‐web/

2 ftp://ccrp.tor.ec.gc.ca/pub/EC_data/AHCCD_daily/
