# Supplementary material for: Northern forest winters have lost cold, snowy conditions that are important for ecosystems and human communities
Source: Ecol Appl. 2019 Aug 7;29(7):e01974. doi: 10.1002/eap.1974 (PMC6851584; doi:10.1002/eap.1974)
Supplement: Supplementary file 1 [file EAP-29-na-s001.pdf]

Supporting Information. Alexandra R. Contosta, Nora J. Casson, Sarah Garlick, Sarah J. Nelson, Matthew P. Ayres, Elizabeth A. Burakowski, John Campbell, Irena Creed, Catherine Eimers, Celia Evans, Ivan Fernandez, Colin Fuss, Thomas Huntington, Kaizad Patel, Rebecca Sanders-DeMott, Kyongho Son, Pamela Templer, and Casey Thornbrugh. 2019. Northern forest winters have lost cold, snowy conditions that are important for ecosystems and human communities. *Ecological Applications.*

Appendix S1

Section S1. Study Area and Data Sources

Although data sources from both the U.S. and Canada underwent extensive quality control prior to public release, we implemented additional pre-processing procedures to 1) screen stations for record completeness both within a given winter and across the measurement period; and 2) gap fill snow data. We defined winter as occurring between November 1 and May 31 to capture both the period of biological dormancy between autumn senescence and spring leaf-out, as well as early and late freezing and snow events across the northern forest study area (Kunkel et al., 2009). The measurement period was defined for the complete record of each variable within a given station, not the 100-year timeframe from 1917 to 2016 applied for initial station selection. Although some of the stations featured data collected prior to 1917, these records tended to have more data gaps and resulted in the entire station not passing record completeness screening.

Stations were evaluated for precipitation, temperature, snowfall, and snow depth record completeness using the following steps applied to each variable in turn. First, winter months missing more than ten days of data were omitted from the analysis. Second, winters lacking more than twenty observations across months were removed. Third, stations containing fewer than 50% complete data over a twenty year timespan within that station's period of record were omitted. These criteria were less stringent than those applied in prior studies that omitted stations

missing more than five days of data within a single winter (e.g., Feng and Hu, 2007), ten days across a winter (e.g., Huntington et al., 2004; Feng and Hu, 2007; Kunkel et al., 2009), and fewer than 50% of stations over a ten-year time period (e.g., Huntington et al., 2004; Feng and Hu, 2007; Vincent et al., 2015). However, using such stringent criteria would have omitted nearly all of the Canadian sites from the analysis, thereby limiting the geographic scope of the study.

Because the period of record completeness differed among measurements recorded at any given site, a station could pass completeness criteria for one variable and fail for another. For example, total daily precipitation might pass, while snowfall might fail. Due to the dearth of stations in the USHCN and the NCDAEC databases that fit within our study area, we did not completely remove a site from the analysis unless both the precipitation and the temperature variables tested within a station failed the completeness criteria. Overall, 37 stations were retained for development and testing of winter climate change indicators. All 37 stations passed record completeness criteria for both precipitation and temperature, such that all 37 stations were included when evaluating winter climate change indicators. Average record completeness for each station was as follows: 79% for precipitation; 74% for daily maximum temperature, 69% for daily minimum temperature, 62% for snowfall, and 27% for snow depth, where record completeness was calculated as the total number of years containing winter records divided by the total number of years spanned by the record for each individual site by variable combination (Table S1). These values were somewhat lower than those reported in prior studies that used stricter completeness criteria (e.g., Huntington et al., 2004). Missing records were distributed evenly across the 100-year timeframe from 1916 to 2017 and were not more frequent earlier in the time series.

Section S2. Modeling Snow Depth and Snowfall

Snow depth and snowfall data were estimated for the entire dataset using a degree-day snowmelt model (Kokkonen et al., 2006; Buttle, 2009; Crossman et al., 2016) implemented in R (R Core Team, 2017) using the *snow.sim* function within the *hydromad* package (Andrews et al., 2011). This model requires two driving variables: daily precipitation (mm d^{-1}) and air temperature (°C). The model simulates snowfall by assuming that above a threshold temperature (T_{max}), all precipitation falls as rain, and below a threshold temperature (T_{min}) it falls as snow, where T_{max} must be greater than T_{min}. It also simulates a snowmelt rate (k_d in mm d^{-1}) as occurring above a threshold temperature (where $T_{melt} = T_{min}$). Temperature thresholds and snowmelt rates were allowed to vary among different stations. Additional parameters can include a degree day factor for snow freezing (k_f), liquid storage capacity of the snowpack (r_{cap}), and correction factors for rain (c_r) and snow (c_s) to account for precipitation gage undercatch. These latter parameters (k_f, r_{cap}, c_r, c_s) were set to zero. The model can perform equally well without them in simulating snow water equivalent (SWE), and their omission reduces the overall complexity of the calibration step (Kokkonen et al., 2006).

Implementation of the model required calibration of three parameters: a rainfall temperature threshold (T_{max}), a snowfall temperature threshold (T_{min}), and a melt rate (k_d). When selecting parameter values, T_{max} and T_{min} were allowed to vary between -2 and 2°C, and k_d ranged from 1 to 4 mm d^{-1} based on prior values published in the literature (e.g., Kokkonen et al., 2006). Since data from weather stations were reported as integers, the step increment for all parameter values was 1. Because T_{max} has to be greater than T_{min} for the model to function, this resulted in 50 possible unique parameter combinations of T_{max}, T_{min}, and k_d for calibrating *snow.sim*. Prior implementation of the model has contained greater numbers of parameter

combinations (e.g., Kokkonen et al., 2006). However, such simulations have included step increments as small as 0.05 for data collected to at least two significant digits, which would not be appropriate for our data set. For each parameter suite, the accumulation of the snowpack as SWE was simulated using the daily mass balance of snowfall minus snowmelt. The modeled snowpack that best matched the data for each weather station was selected by correlating the 50 possible simulated snow depths (in SWE) against the measured snow depth values (in solid snow; e.g., Crossman et al., 2016) and selecting the modeled SWE with the highest r^2 value, which varied from a minimum of 0.05 to a maximum of 0.80 with an average of 0.50. Snow depth values that had been recorded as zero in the historical record were removed prior to the correlation analysis to remove potential bias associated with observer error. Snowfall and rainfall were then simulated using the parameter set of T_{max} and T_{min} that had resulted in the best modeled SWE, as *snow.sim* only outputs total precipitation regardless of whether it falls as rain or snow. Because measured and modeled snow depth and snowfall were not 1:1 analogs (measured were in solid snow, modeled were in SWE), calculations of winter climate change indicators from modeled snow data consisted of presence-absence metrics rather than shifts in amounts of snow cover or snowfall. The model accurately simulated the presence of a snowpack 98% of the time and precipitation falling as snow 95% of the time.

Section S3. Stakeholder Engagement

Woven into the timeline for this project were roundtable dialogue activities with groups of stakeholders intended to elicit a range of perspectives, experiences, observations, and questions related to changing winter climate and associated impacts to people and ecosystems. Led by the Hubbard Brook Research Foundation (HBRF), these "Hubbard Brook Roundtables" involved groups of 10–25 invited participants for one-day facilitated dialogue sessions. Participants were

invited using a snowball method, beginning with the HBRF's existing network of stakeholder advisors. Stakeholder groups represented during these meetings crossed numerous sectors including forestry, logging, land management, conservation, tourism and recreation, rural economic development, wildlife, climate science, environmental education and media, and state and Tribal governments.

The HBRF hosted three separate roundtables from 2016 to 2018: one in Vermont in 2016, one in Maine in 2017, and one in New Hampshire in 2018. For the initial roundtable in Vermont, stakeholders participated in individual, one-hour pre-meeting interviews with the conveners, the notes for which were synthesized into a pre-meeting report that was distributed to the group and served as catalyst for discussion. The HBRF produced summary reports from each event. Many of the indicators of winter climate change calculated in this study were based on insights gained from the roundtable dialogue events about stakeholder interests, concerns, questions, and experiences.

Section S4. Literature Cited

Andrews, F. T., B. F. W. Croke, and A. J. Jakeman. 2011. An open software environment for hydrological model assessment and development. Environmental Modelling and Software 26:1171–1185.

Buttle, J. M. 2009. Using a temperature-based model of snow accumulation and melt to assess the long-term hydrologic behaviour of forested headwater basins in south-central Ontario. 66th Eastern Snow Conference:59–71.

Crossman, J., M. Catherine Eimers, N. J. Casson, D. A. Burns, J. L. Campbell, G. E. Likens, M. J. Mitchell, S. J. Nelson, J. B. Shanley, S. A. Watmough, and K. L. Webster. 2016. Regional meteorological drivers and long term trends of winter-spring nitrate dynamics across watersheds in northeastern North America. Biogeochemistry 130:247–265.

Feng, S., and Q. Hu. 2007. Changes in winter snowfall/precipitation ratio in the contiguous United States. Journal of Geophysical Research Atmospheres 112:D15109.

Huntington, T. G., G. A. Hodgkins, B. D. Keim, and R. W. Dudley. 2004. Changes in the proportion of precipitation occurring as snow in New England (1949-2000). Journal of Climate 17:2626–2636.

Kokkonen, T., H. Koivusalo, T. Jakeman, and J. P. Norton. 2006. Construction of a degree-day snow model in the light of the ten iterative steps in model development. In: Voinoc, A., A. J. Jakeman, A. E. Rizzoli, eds. Proceedings of the iEMSs third biennial meeting: "summit on environmental modelling and software." International Environmental Modelling and Software Society, Burlington, UK.

Kunkel, K. E., M. Palecki, L. Ensor, K. G. Hubbard, D. Robinson, K. Redmond, and D. Easterling. 2009. Trends in twentieth-century U.S. snowfall using a quality-controlled dataset. Journal of Atmospheric and Oceanic Technology 26:33–44.

R Core Team. 2017. R: A language and environment for statistical computing. R Foundation for Statistical Computing, Vienna, Austria. URL https://www.R-project.org/.

Vincent, L. A., X. Zhang, R. D. Brown, Y. Feng, E. Mekis, E. J. Milewska, H. Wan, and X. L. Wang. 2015. Observed trends in Canada's climate and influence of low-frequency variability modes. Journal of Climate 28:4545–4560.

Table S1. Period of record and record completeness for precipitation (Prcp) snow depth (Snwd), snowfall (Snwf), daily maximum temperature (Tmax), and daily minimum temperature (Tmin) of 37 stations included in the analysis.

					**Period of Record**					**Record Completeness (%)**				
**Station**	**Lat**	**Lon**	**Elev (ft)**	**Loc**	**Prcp**	**Snwd**	**Snwf**	**Tmax**	**Tmin**	**Prcp**	**Snwd**	**Snwf**	**Tmax**	**Tmin**
120200	41.64	-84.99	1010	IN	1919–2016	1919–2016	1919–2016	1919–2016	1919–2016	88	85	78	86	84
124837	41.61	-86.72	810	IN	1947–2016	1947–2016	1947–2016	1947–2016	1947–2016	84	54	72	88	77
132864	42.86	-91.80	1050	IA	1917–2016	—	1917–2016	1917–2016	1917–2016	74	—	78	78	71
176905	43.66	-70.30	45	ME	1940–2016	1940–2016	1940–2016	1940–2016	1940–2016	96	87	93	97	97
190736	42.22	-71.12	630	MA	1917–2016	1948–2016	—	1917–2016	1917–2016	85	47	—	95	91
200779	43.71	-85.49	930	MI	1917–2016	—	—	1917–2016	1917–2016	85	—	—	71	57
201675	41.96	-85.00	984	MI	1917–2016	1917–2015	1917–2015	1917–2016	1917–2016	79	65	72	86	75
204104	46.47	-90.19	1430	MI	1917–2016	—	1917–2015	1917–2016	1917–2016	75	—	67	81	75
211630	46.71	-92.52	1265	MN	1917–2016	1951–2016	1949–2016	1917–2016	1917–2016	89	23	37	91	84
214106	47.22	-95.20	1490	MN	1917–2016	—	—	1917–2016	1917–2016	76	—	—	57	49
215435	44.89	-93.22	834	MN	1937–2016	1937–2016	1937–2016	1937–2016	1937–2016	99	91	94	99	97
216547	46.67	-94.12	1250	MN	1917–2016	—	1917–2016	1917–2016	1917–2016	75	—	48	70	59
300042	42.76	-73.80	275	NY	1938–2016	1938–2016	1938–2016	1938–2016	1938–2016	97	95	95	97	97
303033	42.46	-79.30	760	NY	1917–2011	—	1920–2009	1917–2011	1917–2011	67	—	61	89	87
305801	40.79	-73.97	130	NY	1917–2016	1917–2016	1917–2016	1917–2016	1917–2016	94	90	98	92	87
308944	44.16	-74.91	1510	NY	1918–2010	1926–2010	1922–2010	1917–2010	1917–2010	51	35	53	55	46
333780	41.30	-81.16	1230	OH	1917–2016	1917–2016	1917–2016	1917–2016	1917–2016	76	45	49	61	59
336118	41.27	-82.62	670	OH	1917–2016	1917–2016	1917–2016	1917–2016	1917–2016	82	70	65	77	77
339312	40.79	-81.92	1020	OH	1917–2016	—	—	1917–2016	1917–2016	92	—	—	85	83
431081	44.47	-73.16	332	VT	1940–2016	1944–2016	1944–2016	1940–2016	1940–2016	99	86	83	97	97
472001	42.69	-90.12	930	WI	1917–2016	—	1917–2016	1917–2015	1917–2015	75	—	62	67	61
473405	44.12	-89.54	1076	WI	1918–2016	1921–2016	1918–2016	1917–2016	1917–2016	65	63	54	78	70
475120	44.66	-90.14	1250	WI	1917–2016	—	1917–2016	1917–2016	1917–2016	69	—	66	58	46
475516	45.89	-89.74	1580	WI	1917–2016	1947–2016	1917–2016	1917–2016	1918–2016	91	29	82	80	64
478027	45.82	-91.89	1100	WI	1917–2016	1931–2016	1917–2016	1917–2016	1917–2016	54	33	45	70	60
478827	43.57	-90.92	1185	WI	1917–2016	—	1917–2016	1917–2016	1917–2016	74	—	42	46	43
5031320	49.60	-95.20	327	MB	1917–2016	—	1917–2016	1917–2016	1917–2016	85	—	88	25	25
5032162	50.20	-96.10	267	MB	1917–2013	—	1917–2013	1917–2013	1917–2013	83	—	84	80	80
6025205	48.80	-92.60	361	ON	1917–2016	—	1917–2016	1917–2013	1917–2013	81	—	81	83	83

Table S1, *contd.*

					Period of Record					Record Completeness (%)				
**Station**	**Lat**	**Lon**	**Elev (ft)**	**Loc**	**Prcp**	**Snwd**	**Snwf**	**Tmax**	**Tmin**	**Prcp**	**Snwd**	**Snwf**	**Tmax**	**Tmin**
6100971	44.60	-75.70	96	ON	1917–2016	—	1917–2016	1917–2016	1917–2016	93	—	93	68	68
6105976	45.40	-75.70	79	ON	1917–2016	—	1917–2016	1917–2016	1917–2016	97	—	97	94	94
6149625	43.10	-80.80	282	ON	1917–2016	—	1917–2016	1917–2016	1917–2016	78	—	79	75	75
68138	41.80	-72.25	650	CT	1917–2016	—	—	1917–2016	1917–2016	65	—	—	71	59
7014290	45.30	-74.10	47	QC	1920–2010	—	1920–2014	1917–2009	1917–2009	40	—	49	47	47
7022160	45.90	-72.50	82	QC	1917–2015	—	1917–2015	1917–2009	1917–2009	65	—	71	38	38
7025440	46.20	-72.60	30	QC	1917–2015	—	1917–2015	1917–2009	1917–2009	68	—	76	61	61
7027283	46.20	-70.70	168	QC	1917–2015	—	1917–2015	1917–2013	1917–2013	82	—	90	61	61

Table S2. Summary of statistics demonstrating change over time in four snowmaking opportunity indicators that represent two temperature thresholds (-2 °C and -5 °C) and two time periods, prior to December 25 and prior to February 28. Median (Med Slope) and range (Range of Slopes) of trends over time (days/decade) were calculated from Sen slopes in sites where trends were significant (α=0.05), and # Pos and # Neg indicate number of significant positive and negative trends, respectively. Regional trends (Reg Slope) were determined using Sen slope analyses; "ns" indicates lack of significance. Statistics are reported for each of three sub-regions.

	West (n = 15)					Central (n = 9)					East (n = 13)				
Indicator Name	# Pos	# Neg	Med Slope	Reg Slope	Range of Slopes	# Pos	# Neg	Med Slope	Reg Slope	Range of Slopes	# Pos	# Neg	Med Slope	Reg Slope	Range of Slopes
Snowmaking Day (-2°C before Feb 28)	1	5	-0.6	-0.3	-1.4, +0.7	2	2	-0.1	ns	-1.9, +1.4	0	11	-1.4	-1.5	-2.3, -0.9
Snowmaking Day (-2°C before Dec 25)	0	9	-1.2	-0.8	-1.6, -0.8	2	3	-0.9	ns	-2.4, +1.5	0	10	-2.0	-1.7	-2.7, -1.0
Snowmaking / Mosquito Kill Day (-5°C before Feb 28)	0	6	-0.9	-0.5	-1.6, -0.8	1	1	+0.1	ns	-1.3, +1.4	0	12	-1.5	-1.6	-2.7, -1.1
Snowmaking Day (-5°C before Dec 25)	0	9	-1.5	-0.9	-2.2, -0.9	2	0	+1.4	ns	+1.1, +1.8	0	11	-1.6	-1.6	-3.1, -1.0

Table S3. Summary of statistics demonstrating change over time in winter climate change indicators. Median (Med Slope) and range (Range of Slopes) of trends over time (days/decade) are calculated from Sen slope statistics of sites where trends were significant (α=0.05), and # Pos and # Neg indicate number of significant positive and negative trends, respectively. Regional trends (Reg Slope) were determined using Sen slope analyses. Total number of sites = 37.

Indicator Name	# Pos	# Neg	Med slope	Reg Slope	Range of slopes
Thaw Day	11	6	1.1	0.3	-1.6, +2.2
Ice Day	1	26	-1.4	-0.4	-3.8, +1.2
Frost Day	8	11	-1.1	-1.1	-2.3, +2.0
Extreme Cold / Pine Beetle Kill Day	3	15	-1.3	-0.3	-2.2, +1.2
Hemlock Wooly Adelgid Kill Day	0	12	-0.3	0.0	-1.1, 0.0
Snowmaking / Mosquito Kill Day (-5°C annually)	1	19	-1.3	-0.8	-2.7, +1.4
Snow Covered Day	2	16	-1.9	-0.9	-4.5, +1.9
Bare Ground Day	12	1	2.0	0.7	-2.8, +2.8
Rain-on-Snow Day	4	4	0.0	0.0	-0.7, 0.2
Bare Ground Ice / Frozen Ground Day	24	1	1.0	-0.3	-2.0, +3.0
Bare Ground Thaw / Mud Day	15	1	1.7	0.7	-1.9, +2.6
